# Enterovirus D68-Associated Acute Respiratory Illness ─ New Vaccine Surveillance Network, United States, July–November 2018–2020

**DOI:** 10.15585/mmwr.mm7047a1

**Published:** 2021-11-26

**Authors:** Melisa M. Shah, Ariana Perez, Joana Y. Lively, Vasanthi Avadhanula, Julie A. Boom, James Chappell, Janet A. Englund, Wende Fregoe, Natasha B. Halasa, Christopher J. Harrison, Robert W. Hickey, Eileen J. Klein, Monica M. McNeal, Marian G. Michaels, Mary E. Moffatt, Catherine Otten, Leila C. Sahni, Elizabeth Schlaudecker, Jennifer E. Schuster, Rangaraj Selvarangan, Mary A. Staat, Laura S. Stewart, Geoffrey A. Weinberg, John V. Williams, Terry Fan Fei Ng, Janell A. Routh, Susan I. Gerber, Meredith L. McMorrow, Brian Rha, Claire M. Midgley

**Affiliations:** ^1^Division of Viral Diseases, National Center for Immunization and Respiratory Diseases, CDC; ^2^Epidemic Intelligence Service, CDC; ^3^General Dynamics Information Technology, Inc., Falls Church, Virginia; ^4^Baylor College of Medicine, Houston, Texas; ^5^Texas Children’s Hospital, Houston, Texas; ^6^Vanderbilt University Medical Center, Nashville, Tennessee; ^7^Seattle Children’s Hospital, Seattle, Washington; ^8^University of Rochester School of Medicine and Dentistry, Rochester, New York; ^9^Children’s Mercy Hospital, Kansas City, Missouri; ^10^Children’s Hospital of Pittsburgh, University of Pittsburgh Medical Center, Pittsburgh, Pennsylvania; ^11^Department of Pediatrics, Division of Infectious Diseases, Cincinnati Children’s Hospital Medical Center, Cincinnati, Ohio.

Enterovirus D68 (EV-D68) is associated with a broad spectrum of illnesses, including mild to severe acute respiratory illness (ARI) and acute flaccid myelitis (AFM). Enteroviruses, including EV-D68, are typically detected in the United States during late summer through fall, with year-to-year fluctuations. Before 2014, EV-D68 was infrequently reported to CDC (*1*). However, numbers of EV-D68 detection have increased in recent years, with a biennial pattern observed during 2014–2018 in the United States, after the expansion of surveillance and wider availability of molecular testing. In 2014, a national outbreak of EV-D68 was detected (*2*). EV-D68 was also reported in 2016 via local (*3*) and passive national (*4*) surveillance. EV-D68 detections were limited in 2017, but substantial circulation was observed in 2018 (*5*). To assess recent levels of circulation, EV-D68 detections in respiratory specimens collected from patients aged <18 years* with ARI evaluated in emergency departments (EDs) or admitted to one of seven U.S. medical centers^†^ within the New Vaccine Surveillance Network (NVSN) were summarized. This report provides a provisional description of EV-D68 detections during July–November in 2018, 2019 and 2020, and describes the demographic and clinical characteristics of these patients. In 2018, a total of 382 EV-D68 detections in respiratory specimens obtained from patients aged <18 years with ARI were reported by NVSN; the number decreased to six detections in 2019 and 30 in 2020. Among patients aged <18 years with EV-D68 in 2020, 22 (73%) were non-Hispanic Black (Black) persons. EV-D68 detections in 2020 were lower than anticipated based on the biennial circulation pattern observed since 2014. The circulation of EV-D68 in 2020 might have been limited by widespread COVID-19 mitigation measures; how these changes in behavior might influence the timing and levels of circulation in future years is unknown. Ongoing monitoring of EV-D68 detections is warranted for preparedness for EV-D68-associated ARI and AFM.

Since 2017, active, population-based, prospective surveillance of EV-D68–associated ARI among patients aged <18 years has been conducted by seven medical institutions in NVSN.^§ ^Respiratory specimens are collected from pediatric patients experiencing ARI (including fever or respiratory symptoms) who are evaluated in EDs or inpatient settings within NVSN. For this study, specimens collected during July–November were tested for EV-D68 using a validated CDC-developed real-time reverse transcription–polymerase chain reaction assay (*5*). EV-D68 testing algorithms differed by site.^¶^ Demographic and clinical data were collected from medical charts or enrollment interviews. This ARI surveillance platform was not designed to capture neurologic outcomes, such as AFM. Detections of EV-D68 in respiratory specimens during July–November in 2018, 2019, and 2020 were assessed by month, site, sex, race/ethnicity, age group, and comorbidities; characteristics were compared by year using univariable chi-square or Wilcoxon rank-sum tests. EV-D68 detections during July–October 2018 have been previously reported (*5*). For comparison with 2019 and 2020 data, 2018 data were reanalyzed to include July–November. Available EV-D68–positive specimens from 2020 were submitted to CDC for sequencing. This activity was reviewed by CDC and was conducted consistent with applicable federal law and CDC policy.**

Provisional data from July–November indicated that 3,546 (2018), 3,769 (2019), and 2,189 (2020) patients with ARI were tested for EV-D68 across NVSN. Despite approximately 40% fewer patients aged <18 years being tested during 2020 than in 2018 and 2019, the percentage with a positive rhinovirus or enterovirus (RV/EV) test result remained similar (range = 37.0%–44.2%) (Table 1). Among all patients aged <18 years with ARI tested during July–November, EV-D68 was detected in 382 of 3,546 (10.8%) in 2018, but in only six of 3,769 (0.2%) in 2019 and 30 of 2,189 (1.4%) in 2020; among patients with positive RV/EV test results, EV-D68 was detected in 24.3%, 0.4%, and 3.6% in 2018, 2019, and 2020, respectively. EV-D68 was detected at all seven sites in 2018, at four sites in 2019 and at six sites in 2020 (Figure). During 2018, the highest number of EV-D68 detections occurred in September, and the timing of detections varied by site (Figure); in 2020, October had the highest number of detections. In 2020, 16 of 30 detections (53.3%) occurred in Kansas City, Missouri. Among 23 EV-D68–positive specimens sequenced from 2020, all were clade D.

**TABLE 1:** Cases of acute respiratory illness and detections of rhinovirus or enterovirus and enterovirus D68 in pediatric* respiratory specimens, by site^†^ and year^§^ — New Vaccine Surveillance Network, United States, July–November 2018, 2019, and 2020

NVSN site	2018			2019			2020		
	Total ARI	RV/EV positive (% ARI)	EV-D68 positive (% RV/EV)	Total ARI	RV/EV positive (% ARI)	EV-D68 positive (% RV/EV)	Total ARI	RV/EV positive (% ARI)	EV-D68 positive (% RV/EV)
**All sites**	**3,546**	**1,569 (44.2)**	**382 (24.3)**	**3,769**	**1,393 (37.0)**	**6 (0.4)**	**2,189**	**841 (38.4)**	**30 (3.6)**
Cincinnati	489	169 (34.6)	56 (33.1)	552	99 (17.9)	0 (—)	468	132 (28.2)	3 (2.3)
Houston	525	156 (29.7)	28 (17.9)	527	183 (34.7)	2 (1.1)	324	81 (25.0)	3 (3.7)
Kansas City	565	306 (54.2)	54 (17.6)	631	282 (44.7)	1 (0.4)	478	244 (51.0)	16 (6.6)
Nashville	673	202 (30.0)	47 (23.3)	611	95 (15.5)	1 (1.1)	168	66 (39.3)	6 (9.1)
Pittsburgh	689	384 (55.7)	96 (25.0)	698	369 (52.9)	0 (—)	331	191 (57.7)	1 (0.5)
Rochester	308	173 (56.2)	63 (36.4)	471	233 (49.5)	0 (—)	181	62 (34.3)	1 (1.6)
Seattle	297	179 (60.3)	38 (21.2)	279	132 (47.3)	2 (1.5)	239	65 (27.2)	0 (—)

**Abbreviations:** ARI = acute respiratory illness; RV/EV = rhinovirus or enterovirus; EV-D68 = enterovirus D68.

* Patients were aged <18 years; the youngest patient included in the analysis was aged 2 days.

^†^ The seven sites were in Cincinnati, Ohio; Houston, Texas; Kansas City, Missouri; Nashville, Tennessee; Pittsburgh, Pennsylvania; Rochester, New York; and Seattle, Washington. EV-D68 testing algorithms differed slightly across sites. Nashville and Pittsburgh sites test all ARI specimens directly for EV-D68; the other five sites (Cincinnati, Houston, Kansas City, Rochester, and Seattle) first test ARI specimens for RV/EV, and then test RV/EV-positive specimens for EV-D68. Because the Nashville and Pittsburgh sites test ARI specimens for RV and EV-D68 but no other EVs, the total number of RV/EV detections might be underestimated. For the Nashville and Pittsburgh sites, the total number of RV/EVs reported represents specimens positive for RV and/or EV-D68.

^§^ Updated data through November provided for direct comparison; preliminary data for July–October 2018 were previously reported. https://www.cdc.gov/mmwr/volumes/68/wr/mm6812a1.htm

Among 30 patients aged <18 years with EV-D68 in 2020, the median age was 5.3 years, 19 (63.3%) were female, and 15 (50%) required inpatient care (one of whom required mechanical ventilation); none of the patients died (Table 2). Nasal congestion or rhinorrhea, cough, dyspnea, or wheezing were reported in >80% of patients. Asthma or reactive airway disease (RAD) were reported in nearly one half (14; 46.7%) of patients in whom EV-D68 was detected. Compared with the same time frame in 2018, when the median age was 2.9 years and 39.3% of patients with EV-D68–positive respiratory specimens were female, those in 2020 were older (p = 0.04) and more frequently female (p = 0.01).

**TABLE 2:** Demographic and clinical characteristics of patients* evaluated for acute respiratory illness who had positive enterovirus D68 test results — New Vaccine Surveillance Network,^†^ United States, July–November 2018, 2019, and 2020

Characteristic	No. (%)		
	2018	2019	2020
**Total**	**382 (100)**	**6 (100)**	**30 (100)**
**Age group, yrs**			
Median (IQR)	2.9 (1.4–5.1)	7.3 (1.5–12.2)	5.3 (1.7–9.0)
<5	284 (74.3)	3 (50.0)	14 (46.7)
5–17	98 (25.7)	3 (50.0)	16 (53.3)
**Sex**			
Female	150 (39.3)	2 (33.3)	19 (63.3)
Male	232 (60.7)	4 (66.7)	11 (36.7)
**Race/Ethnicity**			
Hispanic	53 (13.9)	4 (66.7)	3 (10.0)
Black, non-Hispanic	161 (42.1)	1 (16.7)	22 (73.3)
White, non-Hispanic	125 (32.7)	0 (—)	1 (3.3)
Other, non-Hispanic	42 (11.0)	1 (16.7)	4 (13.3)
Unknown	1 (0.3)	0 (—)	0 (—)
**Comorbidities**			
Asthma or reactive airway disease	139 (36.4)	2 (33.3)	14 (46.7)
Atopy/Allergic condition (excluding asthma)	75 (19.6)	1 (16.7)	10 (33.3)
**Signs/Symptoms**			
Cough	372 (97.4)	6 (100.0)	27 (90.0)
Nasal congestion/Rhinorrhea	324 (84.8)	5 (83.3)	29 (96.7)
Wheezing	317 (83.0)	6 (100.0)	23 (76.7)
Dyspnea	342 (89.5)	6 (100.0)	25 (83.3)
**Hospital status**			
Treated in ED, not admitted	125 (32.7)	1 (16.7)	15 (50.0)
Admitted	257 (67.3)	5 (83.3)	15 (50.0)

**Abbreviation**: ED = emergency department.

* Patients were aged <18 years; the youngest patient included in the analysis was aged 2 days.

^†^ The seven sites were in Cincinnati, Ohio; Houston, Texas; Kansas City, Missouri; Nashville, Tennessee; Pittsburgh, Pennsylvania; Rochester, New York; and Seattle, Washington.

Among 382 patients with EV-D68–positive specimens in 2018, 53 (13.9%) were Hispanic persons, 125 (32.7%) were non-Hispanic White (White) persons and 161 (42.1%) were Black persons. During the study period in 2019, among six patients with EV-D68–positive specimens, one person was Black and four were Hispanic persons. During the study period in 2020, among 30 patients with EV-D68–positive specimens, three (10.0%) persons were Hispanic, one (3.3%) was White, and 22 (73.3%) were Black. This race/ethnicity distribution was observed during the 2020 study period even after the site in Kansas City, Missouri was excluded, which accounted for approximately one half the cases. In contrast, the race/ethnicity distribution of all patients in NVSN sites with RV/EV was similar across all 3 years, with the proportion of Black persons ranging from 35.0% to 38.3%.

## Discussion

Across all study sites, detection of EV-D68 in respiratory specimens collected from patients with ARI remained low during 2019 and 2020, accounting for 0.4% and 3.6% of RV/EV detections, respectively compared with 24.3% of RV/EV detections during 2018. Similar to 2019, EV-D68 represented only 0.3% RV/EV detections among NVSN sites during July–October 2017 (*5*). EV-D68 clade D was detected in 2020, whereas clade B3 was detected among NVSN sites in 2018 (*5*). Because the numbers of EV-D68 detections reported from local and national surveillance both within and outside NVSN during 2014, 2016, and 2018 were higher compared with 2015, 2017, and 2019, a biennial pattern of circulation had been postulated, and high circulation in 2020 was anticipated. Instead, EV-D68 circulation in NVSN in 2020 appeared only slightly higher than that in 2019 and 2017, but notably lower than that in 2018, with some variations in 2020 by site. As reported for other respiratory viruses (*6*), the lower EV-D68 circulation observed in 2020 might reflect interrupted transmission resulting from COVID-19 mitigation measures including wearing a mask, physical distancing, attention to hand hygiene, and school closures. However, the long-term stability of this biennial pattern of EV-D68 circulation was uncertain even before the COVID-19 pandemic (*7*), making the contribution of COVID-19 mitigation measures to low EV-D68 circulation in 2020 unclear. COVID-19 mitigation measures have been theorized to be less effective at reducing RV/EV circulation compared with that of other respiratory virus types because of differences in stability, transmission route, or rates of asymptomatic transmission (*6*). More information is needed to better understand which RV/EV species and types persisted in 2020, and why detections of EV-D68 were limited. Furthermore, the implications for future EV-D68 circulation are unknown, and continued monitoring is needed.

Although overall detections of EV-D68 were low, severe respiratory illness was observed in infected patients aged <18 years during 2019 and 2020, with one half of patients requiring inpatient admission. Approximately one half of the patients aged <18 years with EV-D68–positive respiratory specimens in 2020 had underlying asthma/RAD, which has been previously associated with EV-D68 (*2*). EV-D68–associated severe respiratory illness continues to be a significant medical concern warranting monitoring and preparedness. In addition, EV-D68 is associated with AFM, a rare but debilitating neurologic condition characterized by flaccid limb weakness or paralysis which has been increasingly recognized in recent years.^††^ Similar to the low number of EV-D68–associated ARI cases in 2020 described in this report, national reports of AFM were also low during 2020 (*8*).

Among 36 patients aged <18 years with EV-D68 detected in respiratory specimens in 2019 and 2020, most were Black persons or Hispanic persons. Health disparities by race and ethnicity have been reported previously for multiple respiratory viruses (*9*), and possibly EV-D68 (*10*). Additional years of NVSN data are needed to better understand potential health disparities related to EV-D68 infection. Disparities might arise from multiple factors including differences by race in asthma prevalence,^§§^ differences in access to health care and preventive measures, or higher risk of EV-D68 exposure or severe disease. 

The findings in this report are subject to at least four limitations. First, the results are not representative of the entire year and might underestimate EV-D68 detections. However, this report describes EV-D68 testing during July–November when enterovirus detections are highest in the United States. Second, although NVSN surveillance sites are located across the United States, they might not be representative of all regions nationwide. Third, the inclusion of data for only 3 years as well as the small number of EV-D68 detections in 2020 limited multivariable analyses. Finally, NVSN enrollment was lower in 2020, compared with previous years, and health care–seeking behaviors might have been different because of the COVID-19 pandemic.

Circulation of EV-D68 in 2020 might have been limited by widespread COVID-19 mitigation measures, and changing mitigation measures might influence future EV-D68 circulation patterns. Continued monitoring of EV-D68 circulation is critical to guiding clinical and public health preparedness for both EV-D68–associated ARI and AFM.

**Summary**

**What is already known about this topic?**

Enterovirus D68 (EV-D68) is associated with acute respiratory illness (ARI) and acute flaccid myelitis. Annual U.S. detections of EV-D68 in respiratory specimens vary; biennial circulation was observed during 2014–2018.

**What is added by this report?**

During July–November 2019 and 2020, six and 30 EV-D68 detections, respectively, were identified in children with ARI enrolled in the seven New Vaccine Surveillance Network sites, representing 0.2% and 1.4% of children with ARI; most patients with EV-D68 were Hispanic or Black persons.

**What are the implications for public health practice?**

EV-D68 is an important pediatric pathogen causing respiratory disease. Circulation in 2020 was lower than anticipated; implications for future circulation are unknown. Continued monitoring and characterization of EV-D68 are critical.

**FIGURE:**
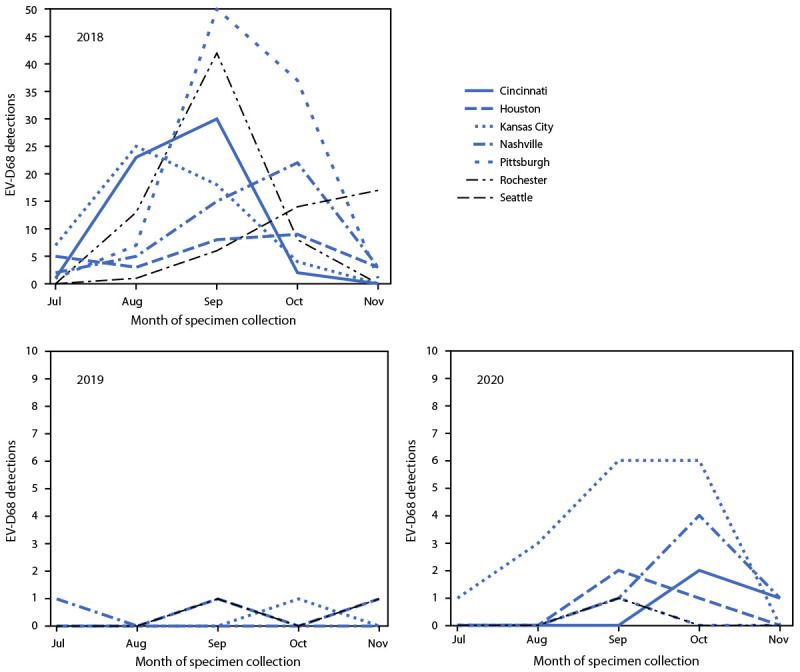
Enterovirus D68 detections, by month and site of specimen collection — New Vaccine Surveillance Network,*^,†^ United States, July–November 2018, 2019, and 2020 **Abbreviation:** EV-D68 = enterovirus D68. * The seven sites were in Cincinnati, Ohio; Houston, Texas; Kansas City, Missouri; Nashville, Tennessee; Pittsburgh, Pennsylvania; Rochester, New York; and Seattle, Washington. ^†^ Only sites with EV-D68 detections during that year are shown. During July–November 2019, there were no EV-D68 detections in Cincinnati, Pittsburgh, or Rochester. During July–November 2020, there were no EV-D68 detections in Seattle.

## Footnotes

* Patients were aged <18 years; the youngest patient included in the analysis was aged 2 days.

† The seven sites were in Cincinnati, Ohio; Houston, Texas; Kansas City, Missouri; Nashville, Tennessee; Pittsburgh, Pennsylvania; Rochester, New York; and Seattle, Washington.

§
https://www.cdc.gov/surveillance/nvsn/index.html

¶ The sites in Nashville and Pittsburgh test all NVSN specimens with a pan-rhinovirus assay (which also detects some enteroviruses because of cross-reactivity) and an EV-D68 assay. The sites in Houston and Rochester conduct a pan-rhinovirus and a pan-enterovirus assay, and if either is positive, a specific EV-D68 assay is conducted. The other three sites (Cincinnati, Kansas City, and Seattle) use a combined rhinovirus/enterovirus assay and if positive, a specific EV-D68 assay is conducted.

** 45 C.F.R. part 46.102(l)(2), 21 C.F.R. part 56; 42 U.S.C. Sect. 241(d); 5 U.S.C. Sect. 552a; 44 U.S.C. Sect. 3501 et seq.

††
https://www.cdc.gov/acute-flaccid-myelitis/index.html

## References

[1] Khetsuriani N, Lamonte-Fowlkes A, Oberst S, Pallansch MA; CDC. Enterovirus surveillance—United States, 1970–2005. MMWR Surveill Summ 2006;55:1–20. 16971890

[2] Midgley CM, Watson JT, Nix WA, ; EV-D68 Working Group. Severe respiratory illness associated with a nationwide outbreak of enterovirus D68 in the USA (2014): a descriptive epidemiological investigation. Lancet Respir Med 2015;3:879–87. 10.1016/S2213-2600(15)00335-5 26482320 PMC5693332

[3] Messacar K, Robinson CC, Pretty K, Yuan J, Dominguez SR. Surveillance for enterovirus D68 in Colorado children reveals continued circulation. J Clin Virol 2017;92:39–41. 10.1016/j.jcv.2017.05.009 28521212 PMC5625344

[4] Abedi GR, Watson JT, Nix WA, Oberste MS, Gerber SI. Enterovirus and parechovirus surveillance—United States, 2014–2016. MMWR Morb Mortal Wkly Rep 2018;67:515–8. 10.15585/mmwr.mm6718a2 29746455 PMC5944979

[5] Kujawski SA, Midgley CM, Rha B, Enterovirus D68–associated acute respiratory illness—new vaccine surveillance network, United States, July–October, 2017 and 2018. MMWR Morb Mortal Wkly Rep 2019;68:277–80. 10.15585/mmwr.mm6812a1 30921299 PMC6448985

[6] Olsen SJ, Winn AK, Budd AP, Changes in influenza and other respiratory virus activity during the COVID-19 pandemic—United States, 2020–2021. MMWR Morb Mortal Wkly Rep 2021;70:1013–9. 10.15585/mmwr.mm7029a1 34292924 PMC8297694

[7] Park SW, Pons-Salort M, Messacar K, Epidemiological dynamics of enterovirus D68 in the United States and implications for acute flaccid myelitis. Sci Transl Med 2021;13:eabd2400. 10.1126/scitranslmed.abd2400 33692131

[8] Kidd S, Yee E, English R, National surveillance for acute flaccid myelitis—United States, 2018–2020. MMWR Morb Mortal Wkly Rep 2021;70:1534–8. 10.15585/mmwr.mm7044a2 34735423 PMC8568096

[9] Iwane MK, Chaves SS, Szilagyi PG, Disparities between black and white children in hospitalizations associated with acute respiratory illness and laboratory-confirmed influenza and respiratory syncytial virus in 3 US counties—2002–2009. Am J Epidemiol 2013;177:656–65. 10.1093/aje/kws299 23436899

[10] Biggs HM, McNeal M, Nix WA, Enterovirus D68 infection among children with medically attended acute respiratory illness, Cincinnati, Ohio, July–October 2014. Clin Infect Dis 2017;65:315–23. 10.1093/cid/cix314 28379349 PMC5708293

